# New-Onset Atrial Fibrillation as an Early Marker of Anthracycline-Associated Cardiotoxicity and Mortality: A Retrospective Study

**DOI:** 10.7759/cureus.108414

**Published:** 2026-05-07

**Authors:** Anna Homeniuk, Gokul Karthikeyan, Anas Atrash

**Affiliations:** 1 Internal Medicine, University of Pittsburgh Medical Center (UPMC), Harrisburg, USA; 2 Internal Medicine, Drexel University College of Medicine, Philadelphia, USA

**Keywords:** anthracycline-induced cardiomyopathy, anthracycline-induced cardiotoxicity, anthracycline toxicity, atrial fibrillation, trinetx

## Abstract

New-onset atrial fibrillation and early readmission after anthracycline initiation may signal a higher risk of mortality and adverse clinical outcomes, but their prognostic value has not been well established. We therefore sought to evaluate this association.

Using the TriNetX federated electronic health record network, we identified adults initiating anthracycline chemotherapy. Among patients with 30-day readmission, we compared those who developed new-onset atrial fibrillation with those who did not. Propensity score matching (1:1) was performed. The outcomes of interest were heart failure hospitalization and all-cause mortality. The final matched cohort included 2,540 patients.

Among patients with 30-day readmission, new-onset atrial fibrillation was associated with significantly worse outcomes than no atrial fibrillation, including higher rates of heart failure hospitalization and all-cause mortality. The subgroup with both new-onset atrial fibrillation and early readmission had the highest cumulative event rates.

New-onset atrial fibrillation during anthracycline therapy, particularly when accompanied by early readmission, identifies a high-risk subgroup for subsequent heart failure hospitalization and death. Incorporating arrhythmia surveillance and post-discharge risk stratification into anthracycline care pathways may improve long-term cardiovascular and survival outcomes in this vulnerable population.

## Introduction

Anthracyclines remain a cornerstone in the treatment of hematologic malignancies and solid tumors, but are limited by well-established cardiotoxicity, traditionally characterized by dose-dependent cardiomyopathy and heart failure [1,2]. However, cardiovascular injury during cancer therapy extends beyond left ventricular dysfunction. Atrial fibrillation is increasingly recognized during active cancer treatment and is independently associated with adverse cardiovascular and survival outcomes in oncology populations [3-5].

Patients with cancer also experience disproportionately high rates of early hospital readmission compared with the general population [6,7], which may reflect underlying treatment-related toxicity and physiologic vulnerability. Despite this, the prognostic significance of new-onset AF and early readmission in patients receiving anthracyclines has not been systematically evaluated. Whether these early clinical events identify a subgroup at heightened risk for subsequent heart failure and death remains unclear. Clarifying this relationship has important implications for surveillance strategies, cardio-oncology referral timing, and targeted intervention, consistent with contemporary cardio-oncology practice recommendations [8,9].

## Materials and methods

We conducted a retrospective cohort study using the TriNetX Global Collaborative Network, a federated electronic health record database including 152 healthcare organizations. The TriNetX platform provides access to de-identified demographic, diagnostic, procedural, and medication data. The Compare Outcomes analytic module was used. 

Adults aged 18 years and older with documented anthracycline exposure were eligible. Anthracycline therapy was defined by ATC class L01DB (anthracyclines and related substances) and by medication records for doxorubicin, daunorubicin, or epirubicin. Atrial fibrillation was defined using ICD-10 code I48, which includes atrial fibrillation and atrial flutter. New-onset atrial fibrillation was operationalized as the first documented I48 diagnosis occurring on or after anthracycline exposure, with no prior atrial fibrillation/flutter diagnosis recorded before anthracycline initiation in the available EHR data. Because this analysis used structured EHR data, the study could not reliably determine whether atrial fibrillation was symptomatic or incidentally detected, nor could it consistently capture the diagnostic modality used, such as routine electrocardiography, inpatient telemetry, ambulatory Holter monitoring, or other rhythm-monitoring tests. The readmission framework required an initial inpatient encounter followed by an inpatient readmission occurring between five days and one month after the first qualifying inpatient event. 

Two mutually exclusive cohorts were constructed. Cohort 1 included patients who received anthracycline therapy and met the 30-day readmission criteria but had no documented atrial fibrillation. Cohort 2 included patients who received anthracycline therapy and met the same readmission criteria, and had new-onset atrial fibrillation, defined as the first atrial fibrillation diagnosis occurring on or after anthracycline exposure. Propensity score matching was performed using the baseline variables available in the TriNetX output. Matching achieved a close balance in age, sex, and race/ethnicity. However, anthracycline indication, cancer stage, treatment intent, baseline comorbidity burden, and validated comorbidity indices such as the Charlson Comorbidity Index were not fully available in the analytic output and therefore could not be incorporated into the matching model.

The index event was derived from cohort definitions as implemented by TriNetX. Outcomes were assessed beginning one day after the index event with no specified end date, thereby capturing all events recorded after cohort entry. The primary outcomes were heart failure hospitalization and all-cause mortality. Heart failure hospitalization/events were defined using a comprehensive ICD-10-CM-based heart failure outcome definition that included hypertensive heart disease with heart failure, systolic heart failure, diastolic heart failure, combined systolic and diastolic heart failure, right heart failure, biventricular heart failure, and unspecified heart failure codes. Therefore, both heart failure with reduced ejection fraction (HFrEF) and heart failure with preserved ejection fraction (HFpEF) were captured when documented by corresponding diagnosis codes. A specific left ventricular ejection fraction (LVEF) cutoff was not applied because LVEF values were not reliably available in the exported TriNetX analytic output. All-cause mortality was identified using the TriNetX “Deceased” demographic status.

To reduce confounding by measured baseline variables, propensity score matching was performed at a 1:1 ratio using the characteristics available in the TriNetX output, including age, sex, race, and ethnicity. Before matching, Cohort 1 included 66,580 patients and Cohort 2 included 2,608 patients; after matching, both cohorts included 2,540 patients. Matching achieved close balance across available baseline demographic characteristics, including age and sex, with a mean current age of 70.2 versus 70.4 years and 43.2% female patients in both cohorts (Table 1). Variables not available in the exported matching output, including formal comorbidity indices, anthracycline indication, cancer stage, treatment intent, baseline LVEF, cumulative anthracycline dose, and cardioprotective medication use, were addressed as limitations, rather than included in the propensity model.

**Table 1:** Baseline characteristics of matched cohorts after 1:1 propensity score matching

Characteristic	Cohort 1 - No AF (n = 2,540)	Cohort 2 - AF (n = 2,540)	Std diff
Age at index, mean ± SD (years)	70.2 ± 14.0	70.4 ± 13.9	0.014
Female	1,098 (43.2%)	1,098 (43.2%)	<0.001
White Patients	2,031 (80.0%)	1,990 (78.3%)	0.040
Black or African American Patients	223 (8.8%)	218 (8.6%)	0.007
Hispanic or Latino Patients	109 (4.3%)	111 (4.4%)	0.004
American Indian or Alaska Native Patients	10 (0.4%)	10 (0.4%)	<0.001
Unknown Race	94 (3.7%)	106 (4.2%)	0.024
Native Hawaiian	10 (0.4%)	23 (0.9%)	0.064
Asian	141 (5.6%)	141 (5.6%)	<0.001
Other Race	38 (1.5%)	59 (2.3%)	0.060

Comparative analyses were performed after matching. TriNetX reported measures of association (risk, risk difference, risk ratio, and odds ratio), Kaplan-Meier survival analyses with log-rank testing and hazard ratios, and number-of-instances analyses with date-grouped event counts. In TriNetX outputs, risk ratios and hazard ratios are presented as Cohort 1 divided by Cohort 2; therefore, values below 1 indicate a higher risk in Cohort 2.

## Results

After propensity score matching, 5,080 patients were included (2,540 per cohort). Matched cohorts were similar in age (mean current age 70.2 vs 70.4 years; mean age at index 64.1 vs 64.4 years) and sex (43.2% female in both cohorts).

Heart failure hospitalization occurred in 302 patients (11.9%) in Cohort 1 (anthracycline plus 30-day readmission without atrial fibrillation) and in 784 patients (30.9%) in Cohort 2 (anthracycline plus 30-day readmission with new-onset atrial fibrillation), corresponding to an absolute risk increase of 19.0% (Table 2).

**Table 2:** Heart failure (HF) and hospitalizations for HF risk analysis between cohorts

Cohort	Patients	Outcome events	Risk
Cohort 1	2,540	302	0.119
Cohort 2	2,540	784	0.309

TriNetX reported a risk difference of -0.190 (95% CI -0.212 to -0.168; p < 0.001) and a risk ratio of 0.385 (95% CI 0.341 to 0.435). Because this risk ratio is calculated as Cohort 1 divided by Cohort 2, it indicates substantially higher heart failure hospitalization risk in the atrial fibrillation cohort (Table 3).

**Table 3:** Comparative risk of heart failure hospitalization between the atrial fibrillation and control cohorts

Measure	Value
Risk difference (95% CI)	-0.190 (-0.212 to -0.168)
Risk ratio (95% CI)	0.385 (0.341 to 0.435)
Odds ratio (95% CI)	0.302 (0.261 to 0.350)
p-value	<0.001

Kaplan-Meier analysis demonstrated marked separation between groups, with a log-rank χ² of 417.93 (p < 0.001). TriNetX reported a hazard ratio of 0.273 (95% CI 0.238 to 0.311) for Cohort 1 relative to Cohort 2, again consistent with a substantially higher hazard of heart failure hospitalization in patients with new-onset atrial fibrillation. Survival probability at the end of follow-up was 65.70% in Cohort 1 versus 21.80% in Cohort 2.

In the number-of-instances analysis, patients with atrial fibrillation experienced more heart failure event instances than those without atrial fibrillation (mean 14.80 vs 7.92; p < 0.001).

All-cause mortality occurred in 1,041 patients (41.0%) in Cohort 1 and 1,469 patients (57.8%) in Cohort 2, representing an absolute risk increase of 16.8% (Table 4).

**Table 4:** Risk analysis for all-cause mortality between cohorts

Cohort	Patients	Outcome events	Risk
Cohort 1	2,540	1,041	0.41
Cohort 2	2,540	1,469	0.578

TriNetX reported a risk difference of -0.169 (95% CI -0.196 to -0.141; p < 0.001) and a risk ratio of 0.709 (95% CI 0.669 to 0.750), indicating higher mortality risk in the atrial fibrillation cohort (Table 5).

**Table 5:** Comparative risk of all cause mortality between the atrial fibrillation and control cohorts

Measure	Value	95% CI	p-value
Risk difference (95% CI)	-0.169	95% CI (-0.196, -0.141)	p < 0.001
Risk ratio (95% CI)	0.709	95% CI (0.669, 0.750)	-
Odds ratio (95% CI)	0.506	95% CI (0.453, 0.566)	-

**Figure 1:**
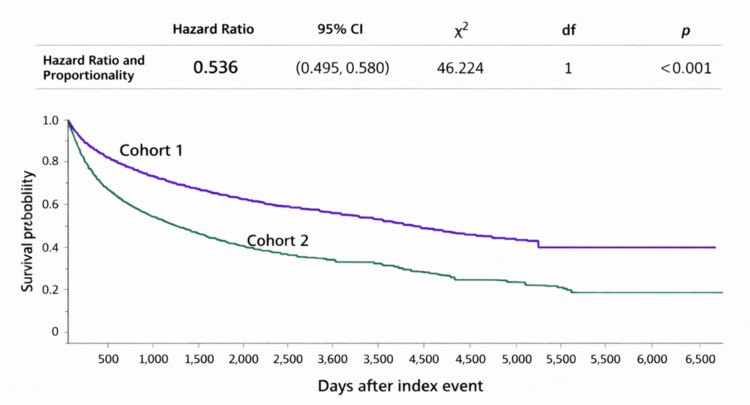
Kaplan-Meier curves demonstrating survival probability in Cohort 1 and Cohort 2

Kaplan-Meier analysis showed significantly worse survival in Cohort 2, with a median survival of 2,047 days in Cohort 1 compared with 438 days in Cohort 2. The log-rank χ² was 244.48 (p < 0.001), and the reported hazard ratio was 0.536 (95% CI 0.495 to 0.580) for Cohort 1 relative to Cohort 2 (Figure 1).​​​​​​

Survival probability at the end of follow-up was 33.97% in Cohort 1 and 16.55% in Cohort 2. 

## Discussion

In this large, propensity-matched, real-world cohort of patients receiving anthracycline therapy and experiencing early hospital readmission, new-onset atrial fibrillation was strongly associated with subsequent heart failure hospitalization and all-cause mortality. Patients with atrial fibrillation had nearly a threefold higher cumulative incidence of heart failure hospitalization and substantially shorter median survival compared with matched patients without atrial fibrillation. These findings suggest that atrial fibrillation in this setting may represent a clinically meaningful marker of heightened cardiotoxic vulnerability rather than an isolated arrhythmic event.

Anthracyclines are well known to cause dose-dependent cardiomyopathy and heart failure through oxidative stress, mitochondrial dysfunction, and topoisomerase IIβ-mediated myocardial injury [1,10]. Landmark analyses by Swain et al. demonstrated the cumulative dose-dependent incidence of congestive heart failure in patients treated with doxorubicin [1], while mechanistic work by Zhang et al. identified topoisomerase IIβ inhibition as central to anthracycline-induced cardiotoxicity [10]. More recently, Cardinale et al. emphasized that early detection and initiation of heart failure therapy improves outcomes in anthracycline-associated cardiac dysfunction [2].

However, cardiovascular injury during chemotherapy is not limited to systolic dysfunction. Contemporary cardio-oncology frameworks recognize arrhythmias, including atrial fibrillation, as frequent and prognostically important complications of cancer therapy [6,9,11,12]. The 2022 ESC Cardio-Oncology Guidelines emphasize that rhythm disturbances may precede or accompany structural cardiac injury and warrant structured monitoring strategies [8].

Anthracycline-associated atrial fibrillation may arise through both indirect and direct mechanisms. Early during chemotherapy, atrial fibrillation may be precipitated by acute systemic stressors such as tumor lysis syndrome, electrolyte disturbances, infection, anemia, volume shifts, or hospitalization-related physiologic stress. These mechanisms may produce transient or reversible atrial fibrillation, particularly in high-risk patients. However, anthracyclines may also promote atrial arrhythmogenesis through direct myocardial and atrial injury, oxidative stress, mitochondrial dysfunction, inflammation, and autonomic/neurohormonal activation, even before clinically overt heart failure develops [4,8,10,12]. Therefore, new-onset atrial fibrillation after anthracycline exposure may reflect both acute reversible triggers and underlying cardiotoxic susceptibility.

Atrial fibrillation occurs more frequently in patients with cancer than in the general population [3-5]. A large population-based analysis by Yun et al. demonstrated significantly increased atrial fibrillation incidence across multiple malignancy types [5]. Farmakis et al. highlighted the bidirectional relationship between cancer and atrial fibrillation, mediated by systemic inflammation, autonomic dysfunction, oxidative stress, and shared comorbidity burden [4]. In oncology populations, atrial fibrillation has been independently associated with thromboembolism, heart failure, and mortality [3,12].

Our findings extend this literature by focusing specifically on anthracycline-exposed patients and integrating early readmission as a clinically relevant risk marker. While prior studies have evaluated atrial fibrillation incidence in cancer more broadly, fewer have examined its prognostic impact within anthracycline-exposed cohorts, and the combined effect of new-onset atrial fibrillation and early readmission remains insufficiently characterized.

Thirty-day readmission among patients with cancer is common and often reflects treatment-related toxicity, comorbidity burden, and clinical instability [6,13]. Prior studies have demonstrated high readmission rates among patients with cancer and associations with adverse outcomes, including mortality and functional decline [13]. In the present analysis, the subgroup with both early readmission and new-onset atrial fibrillation had the highest cumulative event rates, suggesting that this combination may identify patients who warrant closer post-discharge cardiovascular surveillance.

Incorporating atrial fibrillation detection and structured post-discharge risk assessment into anthracycline care pathways may represent a pragmatic opportunity to identify high-risk patients earlier [8,9,14,15]. Clinically, new-onset atrial fibrillation after anthracycline exposure, especially when accompanied by 30-day readmission, may serve as a trigger for earlier cardio-oncology referral, repeat echocardiographic assessment, rhythm monitoring, optimization of cardiovascular risk factors, and consideration of cardioprotective therapy when appropriate.

Several biologically plausible mechanisms may explain the observed association. First, anthracyclines may induce subclinical myocardial injury before overt systolic dysfunction becomes clinically apparent, and atrial fibrillation may represent an early electrical manifestation of this process. Second, cancer-related and chemotherapy-induced inflammatory states may promote atrial structural and electrical remodeling [4,12]. Third, anthracycline-related oxidative stress, mitochondrial injury, and neurohormonal activation may predispose patients to both arrhythmia and subsequent heart failure [10]. Finally, patients who develop atrial fibrillation may have greater baseline cardiovascular vulnerability that is not fully captured by available matching variables. Taken together, atrial fibrillation may function as an early phenotypic expression of cardiotoxic susceptibility rather than a direct causal mediator of all subsequent adverse outcomes. Global longitudinal strain (GLS) may detect subclinical myocardial dysfunction before a decline in LVEF; however, GLS data were not available in the exported TriNetX analytic output. Therefore, we could not determine whether new-onset atrial fibrillation preceded, coincided with, or followed abnormalities in myocardial strain. Future prospective studies incorporating serial echocardiography, including GLS, may help clarify whether atrial fibrillation identifies cardiotoxic vulnerability before imaging evidence of subclinical ventricular dysfunction.

Several limitations warrant consideration. First, the retrospective EHR-based design introduces potential residual confounding despite propensity score matching. Although matching balanced available baseline demographic characteristics, important clinical and oncologic variables, including anthracycline indication, cancer type and stage, treatment intent, performance status, inpatient illness severity, baseline LVEF, pre-existing subclinical cardiac dysfunction, socioeconomic factors, cardioprotective medication use, and validated comorbidity indices such as the Charlson Comorbidity Index, were not fully available in the exported analytic output. These factors may influence both the risk of atrial fibrillation and the risk of subsequent heart failure hospitalization or mortality.

Second, anthracycline cumulative dose, dosing intensity, exposure duration, formulation, and concomitant chemotherapy agents could not be reliably quantified in the exported analytic output. Because anthracyclines are frequently administered as part of combination regimens, including doxorubicin with cyclophosphamide in breast cancer, ifosfamide-containing regimens in sarcoma, and cytarabine-containing regimens in acute leukemia, we could not fully evaluate regimen-specific effects or distinguish anthracycline-associated risk from the broader treatment context.

Third, because heart failure was defined using ICD-10-CM diagnosis codes rather than echocardiographic measurements, we could not classify events according to LVEF-defined categories or apply specific LVEF cutoffs for HFrEF, mildly reduced ejection fraction (HFmrEF), or HFpEF. The analysis also did not fully distinguish incident from recurrent heart failure; therefore, some observed heart failure hospitalizations may represent recurrent or progressive disease rather than entirely new anthracycline-associated cardiotoxicity.

Fourth, the temporal relationship between atrial fibrillation onset and subsequent heart failure hospitalization could not always be established with exact timing granularity using structured EHR documentation. Therefore, the findings should be interpreted as prognostic associations between new-onset atrial fibrillation after anthracycline exposure and subsequent adverse outcomes, rather than definitive evidence that atrial fibrillation directly caused heart failure or death.

Finally, mortality was assessed as all-cause mortality; cancer-specific and cardiovascular-specific mortality were not available. Generalizability may also be influenced by the participating healthcare organizations within the TriNetX network.

## Conclusions

In this large real-world analysis of anthracycline-treated patients experiencing early hospital readmission, new-onset atrial fibrillation was strongly associated with increased heart failure hospitalization and reduced survival. These findings suggest that atrial fibrillation in this setting may represent an early clinical marker of cardiotoxic vulnerability rather than an isolated arrhythmic event.

Incorporating structured arrhythmia surveillance and post-discharge cardiovascular risk assessment into anthracycline care pathways may help identify high-risk individuals earlier and guide timely cardiology involvement. Prospective studies are warranted to determine whether early rhythm monitoring and preemptive cardioprotective strategies can mitigate progression to heart failure and improve long-term outcomes in this vulnerable population. 
